# Visual attention, biological motion perception, and healthy ageing

**DOI:** 10.1007/s00426-018-1068-6

**Published:** 2018-08-07

**Authors:** Hannah C. Agnew, Louise H. Phillips, Karin S. Pilz

**Affiliations:** School of Psychology, University of Aberdeen, William Guild Building, Aberdeen, AB243FX Scotland, UK

**Keywords:** Healthy ageing, Biological motion perception, Visual attention and point-light actions

## Abstract

Biological motion perception is the ability of the visual system to perceive complex human movement patterns. The previous studies have shown a direct link between attentional abilities and performance on biological motion tasks, both of which have been shown to deteriorate with age. However, it is not known whether there is a direct link between age-related deficits in biological motion processing and attention. Here, we investigated whether age-related changes in biological motion perception are mediated by impaired attentional abilities. To assess basic biological motion performance, we asked 42 younger (*M* = 21 years) and 39 older adults (*M* = 69 years) to indicate the facing direction of point-light actions. Performance did not differ between age groups. We assessed visual spatial and selective attentional abilities, using a range of tasks: conjunctive visual search, spatial cueing, and the Stroop task. Across all tasks, older adults were significantly slower to respond and exhibited larger interference/cueing effects, compared to younger adults. To assess attentional demands in relation with biological motion perception, participants performed a biological motion search task for which they had to indicate the presence of a target point-light walker among a varied number of distracters. Older adults were slower, and generally worse than younger adults at discriminating the walkers. Correlations showed that there was no significant relationship between performance in attention tasks and biological motion processing, which indicates that age-related changes in biological motion perception are unlikely to be driven by general attentional decline.

## Introduction

Identifying and recognising the movement of others, also known as biological motion perception, are an important visual ability. Successful decoding of biological motion information provides us with vital cues to social attributes such as mental states, personality traits, and emotions (Bonda, Petrides, Ostry & Evans, 1996; Herbelein, Adolphs, Tranel & Damasio, 2004).

Most commonly, point-light animations are used to study the perception of biological motion. Johansson (1973) first introduced such animations by attaching point lights to the major joints of an actor and filming him walking/running in a dark room. Remarkably, the moving point lights could immediately be recognised as human motion. This perception is achieved by integrating the information from the local point lights into a global percept of a moving figure. A wide range of information can be extracted from point-light displays including information about their gender, identity, and also the emotional state of a person (e.g., Kozlowski and Cutting, 1977; Dittrich, Troscianko, Lea & Morgan, 1996; Vanrie & Verfaille, 2004; Blake & Shiffrar, 2007; Pavlova, 2011).

Many behavioural and neuropsychological studies have shown a direct link between biological motion processing and attention in younger adults (e.g., Thornton, Rensink & Shiffrar, 2002; Battelli, Cavanagh & Thornton, 2003; Pavlova, Birbaumer & Sokolov, 2006; Safford, Hussey, Parasuraman & Thompson, 2010). In fact, various attentional domains have been suggested to influence the processing of biological motion, namely, divided and selective attention. For instance, Thornton, Rensink, and Shiffrar (2002) found that when attention was divided by a demanding secondary task, the discrimination of point-light walkers was significantly disrupted. Likewise, Cavanagh, Labianca and Thornton (2001) demonstrated that the detection of biological motion within a visual search display requires attention. They asked participants to detect the presence of a target walker facing opposite to a varied number of distractor walkers and found that participants had to individually process each walker to determine their facing direction.

Neuroimaging studies have shown that a network of selective attention plays a critical role in biological motion processing. Safford et al. (2010), for example, combined functional magnetic resonance imaging (fMRI) and electroencephalography (EEG) to investigate the relationship between selective attention and biological motion perception. Participants viewed movies consisting of point-light animations that performed both human (e.g., jumping jacks, walking, and kicking) and tool actions (e.g., scissors, hammer, and saw), and were instructed to indicate the repetition of an action. Results showed that when participants had to selectively attend between the different categories, neural activity was highest in the superior temporal sulcus (STS)—a network involved in the processing of biological motion. Importantly, these findings are in line with other neuroimaging studies such as Pavlova, Birbaumer, and Sokolov, (2006) who found that magnetoencephalographic (MEG) responses to biological motion were driven by selective attention. Chandrasekaran and colleagues (2010) further highlighted a relationship between selective attention and biological motion perception. Participants performed a biological motion discrimination task and a range of attention-based tasks such as the Stroop task (Stroop, 1935), the attention network test (ANT; Fan, McCandliss, Sommer, Raz, & Posner, 2005), and a visual search task. It was found that performance on the biological motion task inversely correlated with the amount of interference participants exhibited on the Stroop task, which measures participants’ ability to name congruent and incongruent colour words.

Here, we investigate whether such relationship between biological motion perception and visual attentional abilities also extends into older age. This is important, because both biological motion perception and visual attention have been found to decline with age. Deficits in various domains of visual attention have been found such as temporal (e.g., Georgiou-Karistianis, N., Tang, J., Mehmedbegovic, F., Farrow, M., Bradshaw, J., & Sheppard, D. et al., 2006) and spatial attention (e.g., Lincourt, Folk & Hoyer, 1997), but more importantly, selective attention (e.g., Watson, Maylor & Manson et al., 2002). Common tasks used to measure selective visual attention, for example, are the Stroop task (Stroop, 1935) and visual search tasks. The latter require participants to detect a target in the presence of multiple distractor items. When targets share similar features to distractor items, older adults are significantly slower and less accurate in detecting their targets (e.g., McDowd & Shaw, 2000; Hommel, Li & Li, 2004; Madden & Whiting, 2004). Research on the Stroop task has shown that older adults exhibit greater Stroop interference effects than younger adults for both visual (Stroop, 1935; Hartley, 1993; West & Bell, 1997; West & Alain, 2000; Davidson, Zacks & Williams, 2003; Mutter, Naylor & Patterson, 2005) and auditory versions of the Stroop task (Sommers & Danielson, 1999; Sommers & Huff, 2003).

Results from spatial cueing tasks show that older adults often exhibit larger cueing effects than younger adults (Madden, 1992; Madden, Connolly & Pierce, 1994; Faust & Balota, 1997). However, this is not always the case with some studies finding age equivalence (Folk & Hoyer, 1992; Gottlob & Madden, 1999). Such mixed findings could be related to differences in the type of cues used, i.e., peripheral or central cues (Langley, Kelland Friesen, Saville & Ciernia, 2011). In addition, age-related changes have been found on paradigms assessing temporal attention such as the attentional blink paradigm (Lahar, Issak & McArthur, 2001; Maciokas & Crognale, 2003; Georgiou-Karistianas et al., 2006; Lee & Hsieh, 2007). In that, older adults have been found to show a reduced ability in identifying targets shown in close temporal proximity to each other.

It has also been well documented that biological motion processing changes with age; however, such changes seem to vary based on task and stimuli. For example, research has shown that older adults are impaired at detecting and discriminating point-light walkers in noise (Billino, Bremmer & Gergenfutner, 2008; Pilz, Bennett & Sekular, 2010), need longer stimulus durations to process biological motion as well as younger adults (Norman, Clayton, Shular & Thompson, 2004; Pilz, et al., 2010), and are less accurate at recognising actions or emotions from point-light displays compared to younger adults (Norman, et al., 2004; Insch Bull, Phillips, Allen & Slessor, 2012; Spencer et al., 2016; Agnew et al., 2016). Point-light animations consist of local motion information, the local motion trajectories of the single dots, and global form information, which is revealed when grouping the single dots into a global percept at each point in time. Integrating the local motion information and/or the global form information over time allows the perception of the global motion of the animation. Pilz et al. (2010) asked participants to discriminate the walking direction from computer-generated point-light walkers that contained primarily local motion information, global form information, or both. They found that older adults are less efficient than younger adults at integrating the local motion and global form information of point-light walkers, especially for less familiar stimuli such as inverted point-light walkers. More recently, Spencer et al. (2016) used emotional point-light walkers and found that older adults were less able to discriminate emotions such as sadness or anger, compared to younger adults. Interestingly, though, both older and younger adults were able to recognise emotions based on both local motion and global form information. In contrast, using recorded actions, Agnew et al. (2016) found that older adults had difficulties matching the actions of point-light displays that primarily contained global form information.

The studies cited above clearly highlight that there are age-related differences in both biological motion perception and in various aspects of attention, especially selective attention. To date, however, no studies have explored how they are related. It is likely that age-related changes are not necessarily based on a deficit directly related to biological motion perception but rather on a secondary factor such as attentional task demands. Therefore, this study assesses whether age-related decline in biological motion perception is mediated by impaired attentional abilities. As a measure of biological motion performance, both a facing discrimination and a visual search task with point-light stimuli were employed, similar to a study by Cavanagh et al. (2001). To assess attentional abilities, we employed simple attention-based tasks, which have commonly been used throughout ageing and attention literature. Tasks were similar to the paradigms used in Chandrasekaran et al. (2010), which allowed us to compare our results with their study’s findings. We measured both spatial attention with a spatial cueing task (Posner, 1980), and selective attention using both the Stroop (Stroop, 1935) and a conjunctive visual search task. We anticipated that, overall, older adults would be slower and less accurate at discriminating and detecting the point-light targets on both biological motion tasks, and exhibit larger Stroop interference and spatial cueing effects, compared to younger adults. If indeed, attentional abilities are involved in age-related changes in biological motion perception, we would expect strong relationships between attentional and biological motion tasks.

## Methods

### Participants

42 younger participants (*M* = 21 years; SD = 2.9; range = 18–31; 12 males) and 39 older participants (*M* = 69 years; SD = 7.0; range = 59–83; 9 males) took part in the experiment. Participants were recruited from the student population and the Psychology Participant panel of the University of Aberdeen. All participants were naive as to the purpose of the experiment and satisfied the following visual criteria: normal or corrected to normal visual acuity of at least 20/16 (measured by the Early Treatment Diabetic Retinopathy Study logarithmic vision chart; Told, Baratsits, Garhöfer & Schmetterer, 2013), score within the normal range on the Pelli Robson Contrast Sensitivity test (1.5–2.00/2.5; Pelli & Robson, 1988), and no colour vision deficiency (measured by the City University Colour Vision test). In addition, all older participants had visited an ophthalmologist or an optometrist within the past year and were free of glaucoma, strabismus, amblyopia, macular degeneration, or cataracts. Older participants completed the Montreal Cognitive Assessment (MoCA; Nasreddine et al., 2005), a screening measure for mild cognitive impairment. All participants’ scores were within the normal range (range 26–30/30). Younger (*M* = 13.8) and older (*M* = 14.6) adults did not differ significantly in education years [*t*(79) = − 1.0, *p* = 0.320]. Participants were reimbursed for their time with £5/hour or course credit. Informed consent was received from each participant. The experiment was approved by the local ethics committee and experiments were conducted in accordance with the Declaration of Helsinki.

### Apparatus

The experiment was conducted on an Apple Mac Mini with MATLAB under the Psychtoolbox extension (Brainard, 1997; Pelli, 1997; Kleiner, Brainard, Pelli, Ingling, Murray & Broussard, 2007). Stimuli were presented on a 19 in CRT Dell monitor (model M993S), with a resolution of 1024 × 768 pixels and a refresh rate of 100 Hz. Participants were seated in a darkened room at a distance of approximately 52 cm and viewed the stimuli binocularly.

### General procedure

All participants were tested on an individual basis in a controlled laboratory setting. The experiment consisted of a large battery of tasks, all of which were computer based. The tasks are detailed separately below. Participants completed a biological motion facing direction discrimination task (facing task), a biological motion target detection task (target detection task), and a range of attention tasks (conjunctive visual search, Stroop, and spatial cueing task). The order of tasks was counterbalanced for each participant. Participants were tested in a single 2-h session which included a number of breaks. On all tasks, a standard QWERTY computer keyboard was used to record participants’ responses, in which they were always instructed to respond quickly and to avoid making errors.

### Battery of tasks and procedure

#### Biological motion facing direction discrimination task

**Fig. 1:**
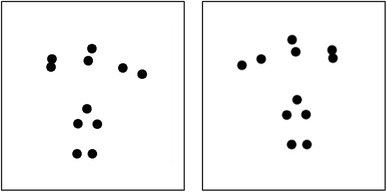
Still frames of rightward (left) and leftward (right) facing point-light actions playing tennis (Vanrie & Verfaille, 2004)

The facing task was employed as a measure of baseline biological motion perception. Stimuli for the facing task consisted of point-light actions playing tennis recorded by Vanrie and Verfaille (2004; Fig. 1). Point-light actions consisted of thirteen dots that simulated points on the head, near the shoulders, both elbows, both wrists, the hip, both knees, and both ankles. Point-light actions depicted only one action: an actor playing an over-arm tennis serve. The actor was viewed at a 90° angle, facing to the left or right from the viewer. Each action subtended a visual angle of 3.0° × 6.0° and each stimulus was presented for 200 ms at a frame rate of 40 frames per second (fps). The stimulus duration was chosen based on the results from an earlier study by Pilz et al. (2010). Point-light actions did not translate across the screen and were presented in the centre of the screen with a 15-pixel jitter along horizontal and vertical that was randomly chosen for each action presentation.

During the experiment, leftward or rightward facing actions were presented on the screen with an inter-stimulus duration of 500 ms. Participants had to indicate the facing direction of the point-light actions by pressing a key (*X* for left and *M* for right). For the main experiment, participants performed one block of 60 trials. Percentage accuracy and reaction times were used as the dependent measures, and we performed correlations between tasks based on both measures.

#### Biological motion target detection task

**Fig. 2:**
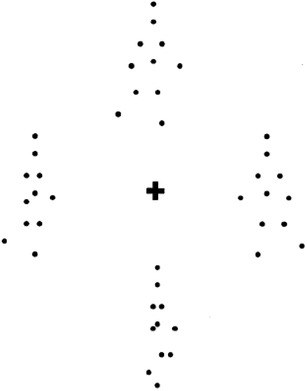
Static frame of a single trial with four walkers. The image depicts a trial, where the target is walking to the left; in this case, the figure on the right-hand side. Participants have to identify the leftward walking target amongst three rightward walking distracters

The stimuli and procedure for the target detection task were adapted from Cavanagh, Labianca, and Thornton (2001; Fig. 2) and a full description of the task can be found in their paper. Here, this task allowed us to directly assess attentional demands related to biological motion perception. Stimuli were point-light walkers generated using a modified version of Cutting’s classic point-light walker algorithm (Cutting, 1978).

The animated walker consisted of 11 dots that simulated points on the head, near the shoulders, both elbows, both wrists, the hip, both knees, and both ankles. In addition, the dots were always visible even when the walker’s body occluded them. The walkers did not translate across the screen, but rather appeared to walk in place with either a rightward or leftward gait. The walker figure subtended a visual angle of 4° × 2° (maximum stride width). The starting point of the walker’s stride and position was selected randomly around fixation on each trial. When more than one walker was displayed, the starting point of the stride cycle for each was randomly chosen and spaced equally around fixation (Cavanagh, Labianca & Thornton, 2001). A complete stride cycle was achieved after 1.3 s. Stimuli were presented on a grey background in the centre of the screen.

At the start of the experiment, participants were instructed as to whether their target walker was facing rightward or leftward. The target facing direction was fixed for each participant, but randomised across participants. Each trial began with a 200 ms presentation of a small black fixation cross. On present trials, this was followed by the predetermined target walker and 1, 2, or 3 distractor walkers (presented with the opposite gait to the target walker), and on absent trials, 2, 3, or 4 distractor walkers were shown. Note, Cavanagh et al. (2001) also included trials, where only one point-light walker was presented. Participants had to identify whether the target walker was present or absent by pressing a key (*M* for present and *X* for absent). Participants had up to 5 s to respond. The duration of the inter-trial interval was 1 s for each trial. All participants performed 5 practice trials to become familiar with the stimuli and task. Feedback was provided during the practice trials but not during the main experiment. For the main experiment, participants performed two blocks of 80 trials, totalling 160 trials. The dependent variables were percentage accuracy, reaction times, and search slopes. Correlational analysis was conducted on reaction time and accuracy performance scores for the largest set size (4), because these scores provided the highest level of variability across participants.

#### Stroop task

Selective attention was assessed using the Stroop task (1935). Four colour words (red, blue, yellow, and green) and eight neutral words (egg, watch, star, fence, poster, door, folder, and dog) in 34-point Helvetica bold font were used as stimuli. Each word was displayed in one of the four different colours (red, blue, yellow, or green). The visual angle of the stimuli varied depending on word length; however, the height of the stimuli subtended to a visual angle of 0.8° and the width ranged between 2.2° and 4.5°. There were three conditions: congruent, incongruent, and neutral. In the congruent condition, the colour words were printed in their corresponding ink colour, whereas in the incongruent condition, the colour words were printed in a mismatched ink colour. In the neutral condition, the neutral words were printed in any of the four different colours. Stimuli were presented on a black background in the centre of the screen.

During the Stroop task, participants were instructed to name the ink colour of the stimulus word whilst ignoring its semantic meaning, by pressing one of the four keys with coloured stickers on (*C* for blue, *V* for red, *B* for green, and *N* for yellow). Stimuli remained on the screen until response.

In the experiment, participants performed one block of trials, in which there were 56 trials per neutral condition, 28 trials in both congruent and incongruent condition, and a total of 112 trials. Stimuli were randomised, so that neither the same word, nor two colour words with the same colour were presented in two consecutive trials. Furthermore, the neutral stimuli were matched with the colour words for word length and were chosen, so that they did not begin with the same letter of any of the colour words. All three trial conditions were randomised within the block. Percentage accuracy and reaction times were used as the dependent measures. A measure of Stroop interference (incongruent trials—congruent trials trials) was computed. Correlations were conducted on this interference measure.

#### Spatial cueing task

**Fig. 3:**
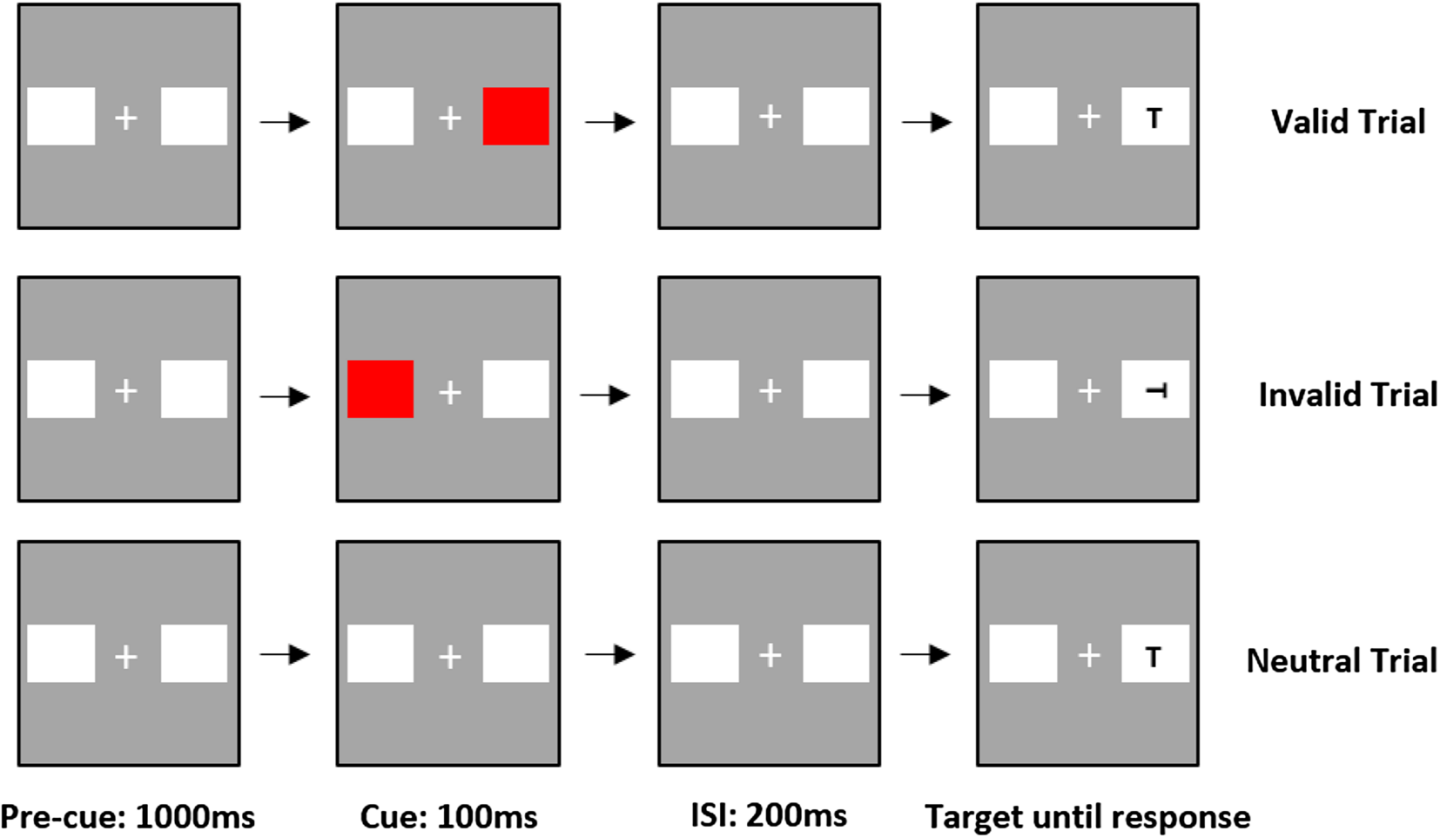
Schematic diagram of single trials for all three cue conditions (valid, invalid, and neutral), for the spatial cueing task. Participants have to identify the target letter; in this case, the target letter is “T”

The stimuli for the spatial cueing task were adapted from Posner (1980) and were used to assess spatial attention. An illustration of the experimental paradigm is shown in Fig. 3. Stimulus displays composed of two dark grey rectangles (each 1.8° × 1.8°) and a black fixation cross (0.45° × 0.45°) located in the centre of the screen. The rectangles were positioned at either side of the fixation cross. The inner edges of the two rectangles were separated by 5° and the fixation cross was positioned 2.5° from the inner edge of the rectangle. Stimulus displays were presented on a light grey background. The two rectangles were presented on screen for 100 ms at the start of the trial. A peripheral flash cue was then presented, which consisted of one of the rectangles briefly filling in red. The cue was presented for 100 ms, and the onset of the target display occurred 200 ms after the onset of the cue. The targets were either the letter “L” or “T”, presented with equal probability in an upright orientation or rotated at 90°, 180°, and 270°. The target letters subtended a visual angle of 0.6° × 0.6°. Finally, the target could either appear in the rectangle, where the preceding cue had occurred (valid), or in the rectangle, where no cue had occurred (invalid). In addition, there were neutral trials in which the target was not preceded by a cue (Neutral).

Participants performed a target discrimination task, in which they were asked to identify the target letter by pressing a key (*S* for “T” and *L* for “L”). The keys were labelled accordingly. Stimuli remained on the screen until response.

In the experiment, each participant performed one block of 192 trials: 120 (75%) valid trials, 36 (12.5%) invalid trials, and 36 (12.5%) neutral trials. All participants performed ten practice trials to familiarise themselves with the stimuli and task. Feedback was provided during the practice trials but not during the main experiment. All three cue types (valid, invalid, and neutral) were randomised within the block.

Percentage accuracy and reaction times were used as the dependent measures. A measure of attentional shifting (invalid trials—valid trials) was calculated. Correlational analysis was conducted on these calculations.

#### Conjunctive visual search task

**Fig. 4:**
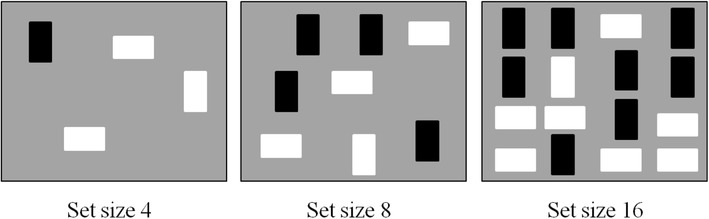
Examples of target-present trials for each of the set sizes (4, 8, and 16) in the conjunctive feature search task. The target item is a white vertical rectangle presented among 3–15 distractor items (black vertical and white horizontal rectangles)

Selective attention was assessed using this conjunctive visual search task. The stimuli were rectangles defined along two features; colour (black or white) and orientation (vertical or horizontal)were presented on a grey background. Examples of the stimuli can be seen in Fig. 4. Stimuli were presented in the centre of the screen within a 4 × 4 matrix, which subtended a visual angle of 10.3° × 10.3°. Stimulus arrays consisted of 4, 8, or 16 randomly positioned items. Each rectangle subtended a visual angle of 0.6° × 1.3°. The target always shared one of the two visual features of the distracters (colour and orientation). There were two conditions: target present and target absent.

The visual search task was separated into four blocks; one block for each target. Participants were asked to indicate whether the target was present or absent by pressing a key (*M* for present and *X* for absent). Stimuli remained on the screen until response.

In the experiment, participants performed four blocks of trials, one for each target, in which the order of the blocks was randomised for each participant. There were 120 trials per block and a total of 480 trials. The dependent variables were percentage accuracy, reaction times, and search slopes. Correlations were conducted on reaction time and accuracy performance scores for the largest set size (16), because these scores provided the highest level of variability across participants.

## Results

### Visual acuity and contrast sensitivity

An independent samples *t* test showed no significant differences in visual acuity between younger (*M* = 1.9, SD = 0.27) and older adults [*M* = 1.1, SD = 0.22; *t*(79) = 1.5, *p* = 0.129, *d* = 3.0]. However, an independent samples *t* test revealed that contrast sensitivity was significantly worse for older (*M* = 1.7, SD = 0.12) compared to younger adults (*M* = 2.0, SD = 0.17; *t*(79) = 4.7, *p* = < 0.001, *d* = 2.0). However, it should be noted that all older adults were above the cutoff of 1.35 on the Pelli Robson Contrast Sensitivity test.

### Biological motion facing direction discrimination task

**Fig. 5:**
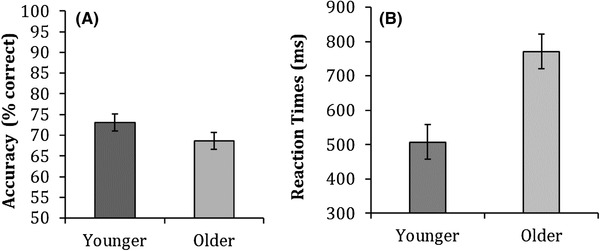
Mean accuracy (**a**) and means of the median reaction times (**b**) for younger and older adults on the facing direction task. Error bars represent ± 1 standard error

Figure 5 shows mean accuracy and median correct reaction times for both younger and older adults on the facing direction task.

Independent samples *t* tests revealed that older adults (*M* = 68, SD = 17.0) performed equally well as younger adults (*M* = 72, SD = 15) at identifying the facing direction of the point-light actions [*t*(79) = 1.2, *p* = 0.235, *d* = 0.3]. However, older adults (*M* = 770, SD = 35) were significantly slower compared to younger adults (*M* = 510, SD = 17) at responding to the point-light actions [*t*(79) = − 4.3, *p* < 0.001, *d* = 0.9].

### Biological motion target detection task

#### Accuracy

**Fig. 6:**
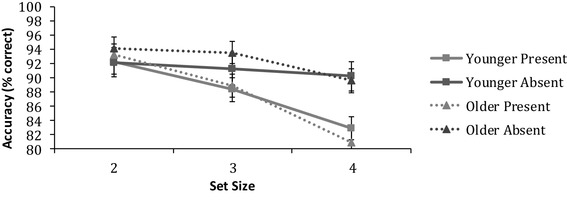
Mean accuracy as a function of set size for both younger and older adults on the biological motion target detection task. Error bars represent ± 1 standard error. Note that due to high-accuracy levels, error bars are very small

Figure 6 displays mean accuracy for younger and older adults on the target detection task. A 2 (age) × 2 (trial condition − present or absent) × 3 (set size) ANOVA revealed a significant main effect of set size [*F* (2,158) = 72.2, *p* < 0.001, $\eta _{{\text{p}}}^{2}$  = 0.5], which was further qualified by a significant set size × age interaction [*F* (2,158) = 3.3, *p* = 0.039, $\eta _{{\text{p}}}^{2}$ = 0.04]. The overall age difference was biggest at set size 4. There seems to be on average a small advantage at the smaller set sizes for older adults; however, the results were not significant.

#### Reaction times

**Fig. 7:**
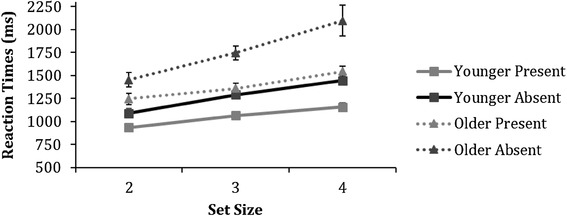
Means of the median reaction times as a function of set size for both younger and older adults on the biological motion target detection task. Error bars represent ± 1 standard error

Figure 7 displays median correct reaction times for younger and older adults on the target detection task. A 2 (age) × 2 (trial condition − present or absent) × 3 (set size) ANOVA showed main effects of *age* [*F* (1,79) = 28.0, *p* < 0.001, $\eta _{{\text{p}}}^{2}$ = 0.3], *trial condition* [*F* (1,79) = 55.0, *p* < 0.001, $\eta _{{\text{p}}}^{2}$ = 0.41], and set size [*F* (2,158) = 71.0, *p* < 0.001, $\eta _{{\text{p}}}^{2}$ = 0.5], which were further qualified by significant interactions for set size × age [*F* (2,158) = 5.0, *p* = 0.008, $\eta _{{\text{p}}}^{2}$ = 0.1] and trial condition × set size [*F* (2,158) = 6.2, *p* = 0.003, $\eta _{{\text{p}}}^{2}$ = 0.1]. The ANOVA revealed no interaction between trial condition × set size × age [*F* (2,158) = 1.5, *p* = 0.226, $\eta _{{\text{p}}}^{2}$ = 0.02].

Post-hoc independent samples *t* test revealed that in each set size [2: *t*(79) = − 4.2, *p* < 0.001, *d* = 0.9 3: *t*(79) = − 4.1, *p* < 0.001, *d* = 1.1 and 4: *t*(79) = − 5.2, *p* < 0.001, *d* = 1.2] older adults exhibited significantly slower reaction times, compared to younger adults. In addition, all participants were found to be significantly slower at responding in the target-absent trials than the target-present trials across all three set sizes [2: *t*(79) = − 4.5, *p* < 0.001, *d* = 0.5, 3: *t*(79) = − 9.1, *p* < 0.001, *d* = 1.0 and 4: *t*(79) = − 4.6, *p* < 0.001, *d* = 0.5], as shown in a post-hoc paired samples *t* test.

#### Search slopes

**Fig. 8:**
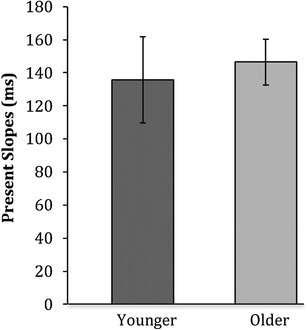
Means of the median search slopes (present trials only) for younger and older adults on the biological motion target detection task. Error bars represent ± 1 standard error

Linear search slopes (reaction times × set size) were calculated (Fig. 8). Independent samples *t* tests revealed that search slopes did not differ between younger (*M* = 140, SD = 17) and older (*M* = 150, SD = 9) adults, in that both groups were as efficient as each other at searching for the point-light walker targets [*t*(79) = − 0.353, *p* = 0.725, *d* = 0.1].

### Conjunctive visual search task

#### Accuracy

**Fig. 9:**
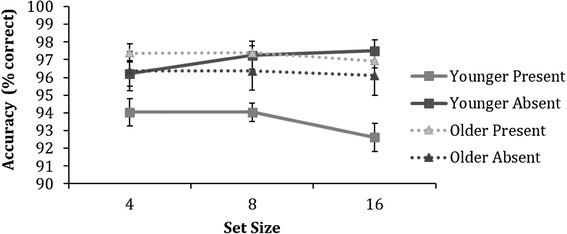
Mean accuracy as a function of set size for both younger and older adults on the conjunctive visual search task. Error bars represent ± 1 standard error. Note that due to high-accuracy levels, error bars are very small

Figure 9 shows mean accuracy for younger and older adults on the conjunctive visual search task. A 2 (age) × 2 (trial condition − present or absent) × 3 (set size) ANOVA revealed a significant main effect of age [*F* (1,79) = 4.1, *p* = 0.050, $\eta _{{\text{p}}}^{2}$ = 0.05], a significant trial condition × age interaction [*F* (1,79) = 10.3, *p* = 0.002, $\eta _{{\text{p}}}^{2}$ = 0.1], and a trial condition × set size interaction [*F* (2,158) = 5.9, *p* = 0.004, $\eta _{{\text{p}}}^{2}$ = 0.1]. In addition, a significant trial condition × set size × age was found [*F* (2,158) = 4.3, *p* = 0.015, $\eta _{{\text{p}}}^{2}$ = 0.05]. To further assess this three-way interaction, we carried out 3 separate age × trial condition ANOVAs for each set size condition (4, 8, and 16).

For set size 4, a main effect of age was found [*F* (1,79) = 4.7, *p* = 0.034, $\eta _{{\text{p}}}^{2}$ = 0.06], older adults were overall more accurate than younger adults, and a trial condition × age interaction [*F* (1,79) = 4.4, *p* = 0.039, $\eta _{{\text{p}}}^{2}$ = 0.05]. Post-hoc independent samples *t* test revealed that younger adults were significantly worse than older adults at responding to the target-present trials [*t*(79) = − 3.6, *p* < 0.001, *d* = 0.8], but performed equally well in the target-absent trials [*t*(79) = − 0.171, *p* = 0.865, *d* = 0.04].

For set size 8, only a significant trial condition × age interaction [*F* (1,79) = 8.5, *p* = 0.005, $\eta _{{\text{p}}}^{2}$ = 0.1] was found, but no main effects of age [*F* (1,79) = 2.6, *p* = 0.108, $\eta _{{\text{p}}}^{2}$ = 0.03] or trial condition [*F* (1,79) = 2.2, *p* = 0.142, $\eta _{{\text{p}}}^{2}$ = 0.03]. Similarly, post-hoc independent samples *t* test revealed that both age groups performed on the par in the target-absent trials [*t*(79) = 0.64, *p* = 0.527, *d* = 1.1], but younger adults exhibited decreased performance when responding to the target-present trials, compared to older adults [*t*(79) = − 5.0, *p* < 0.001, *d* = 0.1].

Finally, for set size 16, a main effect of trial condition [*F* (1,79) = 8.3, *p* = 0.005, $\eta _{{\text{p}}}^{2}$ = 0.1] was found, and all participants performed better in the target-absent trials, compared to the target-present trials. In addition, a significant trial condition × age interaction [*F* (1,79) = 16.1, *p* < 0.001, $\eta _{{\text{p}}}^{2}$ = 0.2] was found. Post-hoc independent samples *t* test showed that older adults performed significantly better in the target-present trials than younger adults [*t*(79) = − 4.8, *p* < 0.001, *d* = 1.1]; however, there were no age differences between the groups in the target-absent trials [*t*(79) = 1.1, *p* = 0.269, *d* = 0.2].

#### Reaction times

**Fig. 10:**
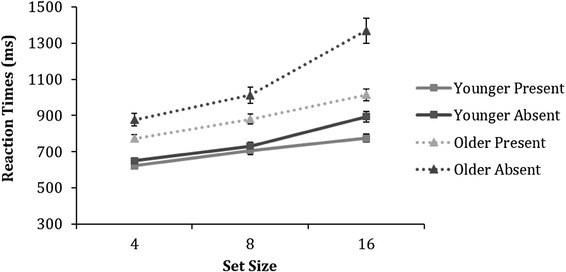
Means of the median reaction times as a function of set size for younger and older adults on the conjunctive visual search task. Error bars represent ± 1 standard error

Figure 10 displays means of the median correct reaction times for younger and older participants on the conjunctive visual search task. A 2 (age) × 2 (trial condition − present or absent) × 3 (set size) ANOVA revealed significant main effects of age [*F* (1,79) = 37.6, *p* < 0.001, $\eta _{{\text{p}}}^{2}$ = 0.3], trial condition [*F* (1,79) = 74.3, *p* < 0.001, $\eta _{{\text{p}}}^{2}$ = 0.5] and set size [*F*(2,158) = 363.2, *p* < 0.001, $\eta _{{\text{p}}}^{2}$ = 0.8] which were further qualified by significant interactions for trial condition × age [*F* (1,79) = 23.6, *p* < 0.001, $\eta _{{\text{p}}}^{2}$ = 0.2], set size × age [*F* (2,158) = 34.2, *p* < 0.001, $\eta _{{\text{p}}}^{2}$ = 0.3] and trial condition × set size [*F* (2,158) = 91.1, *p* < 0.001, $\eta _{{\text{p}}}^{2}$ = 0.5]. Finally, a significant trial condition × set size × age was found [*F* (2,158) = 18.1, *p* < 0.001, $\eta _{{\text{p}}}^{2}$ = 0.2]. To further assess this three-way interaction, we carried out 3 separate age × trial condition ANOVAs for each set size condition (4, 8, and 16).

For set size 4, both a main effect of age was found [*F* (1,79) = 35.3, *p* < 0.001, $\eta _{{\text{p}}}^{2}$ = 0.3], where older adults were significantly slower than younger adults, and trial condition [*F* (1,79) = 37.4, *p* < 0.001, $\eta _{{\text{p}}}^{2}$ = 0.3] as all participants responded faster in the target present than the target-absent trials. In addition, a significant trial condition × age interaction [*F* (1,79) = 13.2, *p* < 0.001, $\eta _{{\text{p}}}^{2}$ = 0.1] was found. Post-hoc independent samples *t* test revealed that older adults were significantly slower at responding in both the target present [*t*(79) = − 5.3, *p* < 0.001, *d* = 1.2] and target-absent trials [*t*(79) = − 6.0, *p* < 0.001, *d* = 1.3], compared to younger adults.

Similarly, in set size 8, both a main effect of age was found [*F* (1,79) = 30.4, *p* < 0.001, $\eta _{{\text{p}}}^{2}$ = 0.3], where older adults were significantly slower than younger adults, and trial condition [*F* (1,79) = 30.3, *p* < 0.001, $\eta _{{\text{p}}}^{2}$ = 0.3] as all participants responded faster in the target present than the target-absent trials. In addition, a significant trial condition × age interaction [*F* (1,79) = 13.2, *p* < 0.001, $\eta _{{\text{p}}}^{2}$ = 0.2] was found. Post-hoc independent samples *t* test revealed that older adults were significantly slower at responding in both the target present [*t*(79) = − 4.8, *p* < 0.001, *d* = 1.1] and target-absent trials [*t*(79) = − 5.7, *p* < 0.001, *d* = 1.2], compared to younger adults.

Finally, in set size 16, both a main effect of age was found [*F* (1,79) = 43.6, *p* < 0.001, $\eta _{{\text{p}}}^{2}$ = 0.4], where older adults were significantly slower than younger adults, and trial condition [*F* (1,79) = 102, *p* < 0.001, $\eta _{{\text{p}}}^{2}$ = 0.6] as all participants responded faster in the target present than the target-absent trials. In addition, a significant trial condition × age interaction [*F* (1,79) = 26.2, *p* < .001, $\eta _{{\text{p}}}^{2}$ = 0.2] was found. Post-hoc independent samples *t* test revealed that older adults were significantly slower at responding in both the target present [*t*(79) = − 6.0, *p* < 0.001, *d* = 1.3] and target-absent trials [*t*(79) = − 6.5, *p* < 0.001, *d* = 1.4], compared to younger adults.

#### Search slopes

**Fig. 11:**
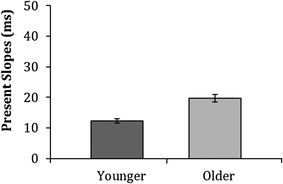
Means of the median search slopes (present trials only) for younger and older adults on the conjunctive visual search task. Error bars represent ± 1 standard error

Linear search slopes (reaction times × set size) were calculated (Fig. 11). Independent samples *t* tests revealed that older adults (*M* = 19, SD = 8) were as efficient at searching for the targets as younger adults (*M* = 12, SD = 5) on the visual search task [*t*(79) = − 1.4, *p* = 0.169, *d* = 0.3].

### Stroop task

**Fig. 12:**
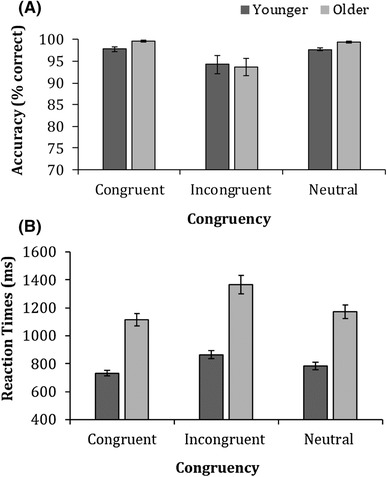
Mean accuracy (**a**) and means of the median reaction times (**b**) for younger and older adults on the Stroop task. Error bars represent ± 1 standard error. Note that due to high-accuracy errors, bars are very small

Figure 12 shows mean accuracy and median correct reaction times for both younger and older adults on the Stroop task. A 2 (age) × 3 (congruency − congruent, incongruent and neutral) ANOVA on accuracy revealed a significant main effect of congruency [*F* (2,158) = 9.3, *p* < 0.001, $\eta _{{\text{p}}}^{2}$ = 0.1], but no main effect of age [*F* (2,158) = 1.1, *p* = 0.302, $\eta _{{\text{p}}}^{2}$ = 0.01]. As expected, overall participants were more accurate at responding to the congruent colour and neutral words, compared to the incongruent colour words. The ANOVA revealed no interaction between congruency × age [*F* (2,158) = 0.683, *p* = 0.507, $\eta _{{\text{p}}}^{2}$ = 0.01].

Furthermore, a 2 (age) × 3 (congruency) ANOVA on reaction times revealed significant main effects of age [*F* (1,79) = 61.1, *p* < 0.001, $\eta _{{\text{p}}}^{2}$ = 0.4] and congruency [*F* (2,158) = 64.1, *p* < 0.001, $\eta _{{\text{p}}}^{2}$ = 0.4] which were further qualified by a significant congruency × age interaction [*F* (2,158) = 7.7, *p* = 0.001, $\eta _{{\text{p}}}^{2}$ = 0.1]. Post-hoc independent samples *t* test revealed that compared to younger adults, older adults were significantly slower to respond across all three conditions [congruent: *t*(79) = − 8.2, *p* < .001, *d* = 1.8, incongruent: *t*(79) = − 7.1, *p* < .001, *d* = 1.6 and neutral: *t*(79) = − 7.2, *p* < .001, *d* = 1.6], compared to younger adults. To establish whether age groups differed in their level of Stroop interference, interference scores were calculated (incongruent RT–congruent RT) for both younger and older adults. Independent samples *t* tests revealed that older adults (*M* = 250, SD = 27) exhibited significantly larger interference effects than younger adults (*M* = 130, SD = 10) on the Stroop task [*t*(79) = − 2.7, *p* = 0.008, *d* = 0.6].

### Spatial cueing task

**Fig. 13:**
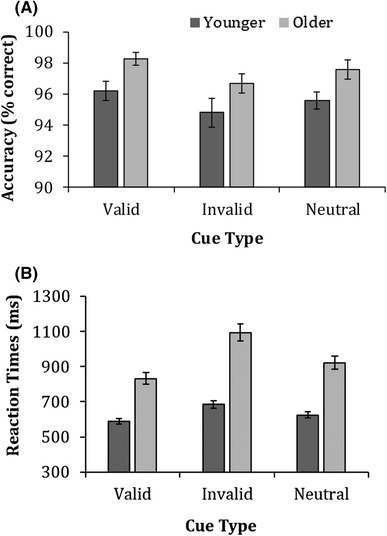
Mean accuracy (**a**) and means of the median reaction times (**b**) for younger and older adults on the spatial cueing task. Error bars represent ± 1 standard error. Note that due to high-accuracy levels, error bars are very small

Figure 13 shows mean accuracy and median correct reaction times for both younger and older adults on the spatial cueing task. A 2 (age) × 3 (cue type − valid, invalid, and neutral) ANOVA on accuracy found significant main effects of *age* [*F* (1,79) = 6.8, *p* = 0.011, $\eta _{{\text{p}}}^{2}$ = 0.8], overall, older adults performed better in all cue conditions than younger adults, and cue condition [*F* (2,158) = 5.0, *p* = 0.008, $\eta _{{\text{p}}}^{2}$ = 0.1], and overall, all participants performed better for valid compared to neutral and invalid trials. The ANOVA revealed no interaction between cue condition × age [*F* (2,158) = 0.009, *p* = 0.991, $\eta _{{\text{p}}}^{2}$ = 0.001].

In addition, a 2 (age) × 3 (cue condition − valid, invalid, and neutral) ANOVA on reaction times revealed significant main effects of age [*F* (1,79) = 57.2, *p* < 0.001, $\eta _{{\text{p}}}^{2}$ = 0.4] and cue condition [*F* (2,158) = 140.3, *p* < 0.001, $\eta _{{\text{p}}}^{2}$ = 0.6] which were further qualified by a significant cue condition × age interaction [*F* (2,158) = 29.6, *p* < 0.001, $\eta _{{\text{p}}}^{2}$ = 0.3]. Post-hoc independent samples *t* test showed that older adults were significantly slower to respond across all cue conditions [valid: *t*(79) = − 6.7, *p* < 0.001, *d* = 1.5, invalid: *t*(79) = − 7.8, *p* < 0.001, *d* = 1.7 and neutral: *t*(79) = − 7.5, *p* < 0.001, *d* = 1.6], compared to younger adults. To establish whether cueing effects differed in magnitude between age groups, cueing scores were calculated (invalid RT − valid RT). Independent samples *t* tests revealed that older adults (*M* = 270, SD = 16) exhibited significantly larger cueing effects than younger adults (*M* = 100, SD = 6) on the spatial cueing task [*t*(79) = − 6.4, *p* < 0.001, *d* = 1.4].

### Biological motion processing and attentional abilities

To determine whether there was a relationship between age, biological motion processing, and attentional abilities, Pearson’s correlation coefficients on both reaction time (Tables 1, 2) and accuracy data (Tables 3, 4) were determined between all tasks, separately for younger and older participants. Due to the diversity of the tasks used, we computed singular scores for each task so as to make reaction time/accuracy scores more comparable. These scores, as well as specific task analysis, can be found in methods. In addition, to ensure that our results were not being driven by optical factors, we included visual acuity and contrast sensitivity scores within our correlational analysis. To correct for multiple comparisons, the Benjamini–Hochberg procedure was carried out (Benjamini & Hochberg, 1995).

**Table 1:** Correlations on reaction times between biological motion and attention tasks, visual acuity, and contrast sensitivity for younger participants

Measure	1	2	3	4	5	6	7
1. Facing direction	–	0.002 ***0.989***	0.231 ***0.140***	0.166 ***0.238***	0.089 ***0.374***	0.062 0.***607***	− 0.071 ***0.656***
2. Visual search		–	− 0.097 ***0.542***	− 0.076 ***0.634***	− 0.315 ***0.042***	0.022 0.***891***	0.140 ***0.376***
3. Stroop task			–	− 0.047 ***0.768***	− 0.059 0.***710***	− 0.041 0.***796***	− 0.014 0.***930***
4. Spatial cueing				–	− 0.007 − 0.***963***	− 0.008 0.***959***	− 0.152 ***0.335***
5. Target detection					–	0.070 ***0.661***	0.068 ***0.668***
6. Visual acuity						–	0.464* ***0.002***
7. Contrast sensitivity							–

None of the correlations between the biological motion and attention tasks were significant when using the Benjamini–Hochberg false discovery procedure (Benjamini & Hochberg, 1995)

*p* values are in bold and Italics, *p < 0.0004

With a false discovery rate of 0.1, only significant correlations were found between visual acuity and contrast sensitivity for both younger (*r* = 0.464, *n* = 42, *p* = 0.002) and older adults (*r* = 0.447, *n* = 39, *p* = 0.004). This simply indicates that the better the visual acuity of participants, the better their contrast sensitivity. The remaining *p* values failed to reach the critical value as computed with the Benjamini–Hochberg procedure (Benjamini & Hochberg, 1995), i.e., there were no significant correlations between reaction time, or accuracy across all the biological motion and attention tasks for both age groups (Tables 1, 2, 3, 4). These results indicate that age-related changes in biological motion perception are unrelated to changes in attentional performance.

**Table 2:** Correlations on reaction times between biological motion and attention tasks, visual acuity, and contrast sensitivity for older participants

Measure	1	2	3	4	5	6	7
1. Facing direction	–	0.211 ***0.198***	− 0.110 ***0.504***	0.009 ***0.955***	− 0.061 ***0.710***	− 0.118 ***0.476***	− 0.006 ***0.970***
2. Visual search		–	0.290 ***0.073***	0.278 ***0.067***	− 0.124 ***0.451***	0.093 ***0.572***	0.000 ***0.999***
3. Stroop task			–	0.111 ***0.502***	− 0.168 ***0.307***	− 0.177 ***0.281***	0.059 ***0.721***
4. Spatial cueing				–	0.033 ***0.841***	− 0.066 ***0.691***	− 0.042 ***0.799***
5. Target detection					–	− 0.052 ***0.753***	− 0.260 ***0.110***
6. Visual acuity						–	0.447* ***0.004***
7. Contrast sensitivity							–

None of the correlations between the biological motion and attention tasks were significant when using the Benjamini–Hochberg false discovery procedure (Benjamini & Hochberg, 1995)

*p* values are in bold and Italics, *p < 0.0004

**Table 3:** Correlations on accuracy between biological motion and attention tasks, visual acuity, and contrast sensitivity for younger participants

Measure	1	2	3	4	5	6	7
1. Facing direction	–	0.285 ***0.067***	− 0.044 ***0.780***	− 0.014 ***0.930***	0.264 ***0.092***	− 0.010 ***0.950***	− 0.118 ***0.459***
*2*. Visual search		–	− 0.112 ***0.478***	0.098 ***0.538***	0.384 ***0.012***	− 0.212 ***0.177***	− 0.122 ***0.441***
3. Stroop task			–	0.246 ***0.116***	− 0.205 ***0.193***	0.066 ***0.679***	0.112 ***0.482***
4. Spatial cueing				–	− 0.263 ***0.092***	− 0.038 ***0.810***	0.053 ***0.738***
5. Target detection					–	0.039 ***0.806***	− 0.002 ***0.992***
6. Visual acuity						–	0.464* ***0.002***
7. Contrast sensitivity							–

None of the correlations between the biological motion and attention tasks were significant when using the Benjamini–Hochberg false discovery procedure (Benjamini & Hochberg, 1995)

*p* values are in bold and Italics, *p < 0.0004

**Table 4:** Correlations on accuracy between biological motion and attention tasks, visual acuity, and contrast sensitivity for older participants

Measure	1	2	3	4	S	6	7
1. Facing direction	–	− 0.101 ***0.541***	0.202 ***0.217***	0.222 ***0.174***	**− 0**.011 ***0.948***	**−** 0.024 ***0.884***	0.117 **0**.***476***
2. Visual search		–	**− 0**.150 ***0.361***	0.188 ***0.253***	0.225 ***0.169***	**− 0**.123 ***0.457***	0.039 ***0.811***
3. Stroop task			–	0.383 ***0.016***	0.109 ***0.510***	0.314 ***052***	0.002 ***0.988***
4. Spatial cueing				–	**− 0**.049 0.765	0.046 ***0.783***	**− 0**.332 ***0.039***
5. Target detection					–	0.151 ***0.359***	0.086 0.***602***
6. Visual acuity						–	0.447* ***0.004***
7. Contrast sensitivity							–

None of the correlations between the biological motion and attention tasks were significant when using the Benjamini–Hochberg false discovery procedure (Benjamini & Hochberg, 1995)

*p* values are in bold and Italics, *p < 0.0004

### Inter-task reliability correlations

To assess the inter-task reliability of our biological motion perception and attention measures, each task’s data were split into half and Pearson correlation coefficients were determined between both halves, separately for younger (Table 5) and older participants (Table 6). It is important to note that split-half correlations were only conducted on reaction time data, because these results provided the highest level of variability across participants. Split-half reliability correlations showed significant reliabilities for most conditions in all tasks.

**Table 5:** Split-half reliability correlations on reaction times across for younger participants

Measure	Half A	Half B	*r*
	M(SD)	M(SD]	
Facing direction	0.63 (0.23)	0.62 (0.21)	0.753
Target detection			
Present (2)	1.1 (0.32)	1.0 (0.29)	0.627
Present (3)	1.2 (0.34)	1.1 (0.31)	0.844
Present (4)	1.4 (0.38)	1.2 (0.37)	0.668
Absent (2)	1.2 (0.33)	1.1 (0.36)	0.837
Absent (3)	1.4 (0.42)	1.3 (0.35)	0.750
Absent (4)	1.5 (0.36)	1.4 (0.38)	0.805
Visual search			
Present (4)	0.71 (0.17)	0.68 (0.16)	0.803
Present (8)	0.83 (0.25)	0.78 (0.30)	0.787
Present (16)	0.89 (0.21)	0.85 (0.25)	0.835
Absent (4)	0.77 (0.20)	0.74 (0.20)	0.842
Absent (8)	0.87 (0.23)	0.80 (0.26)	0.681
Absent (16)	1.1 (0.30)	0.97 (0.29)	0.814
Spatial cueing			
Valid	0.63 (0.14)	0.62 (.14)	0.544
Invalid	0.73 (0.16)	0.72 (.16)	0.756
Neutral	0.65 (0.13)	0.71 (.27)	0.814
Stroop task			
Congruent	0.85 (0.29)	0.75 (.17)	0.303*
Incongruent	0.98 (0.26)	0.89 (.22)	0.836
Neutral	0.86 (0.28)	0.82 (.22)	0.877

The correlations showed significant reliabilities for most conditions in all tasks

All correlations significant at *p* < 0.05 except *

**Table 6:** Split-half reliability correlations on reaction times across for older participants

Measure	Half A	HalfB	*r*
	M(SD)	M(SD)	
Facing direction	1.1 (.47)	0.97 (0.36)	0.756
Target detection			
Present (2)	1.4 (0.56)	1.2 (0.32)	0.708
Present (3)	1.6 (0.51)	1.4 (0.34)	0.592
Present (4)	1.7 (0.44)	1.5 (0.33)	0.784
Absent (2)	1.6 (0.47)	1.5 (0.54)	0.450
Absent (3)	1.8 (0.48)	1.7 (0.41)	0.642
Absent (4)	2.1 (0.60)	1.8 (0.35)	0.646
Visual search			
Present (4)	0.89 (0.18)	82 (0.16)	0.826
Present (8)	1.1 (0.37)	.95 (0.25)	0.307*
Present (16)	1.2 (0.41)	1.1 (0.25)	0.703
Absent (4)	1.1 (0.32)	.96 (0.29)	0.682
Absent (8)	1.2 (0.38)	1.1 (0.36)	0.627
Absent (16)	1.6 (0.49)	1.5 (0.63)	0.774
Spatial cueing			
Valid	0.97 (0.34)	.89 (0.26)	0.574
Invalid	1.2 (0.34)	1.1 (0.37)	0.841
Neutral	1.0 (0.28)	.99 (0.30)	0.842
Stroop task			
Congruent	1.3 (0.35)	1.1 (0.30)	0.708
Incongruent	1.6 (0.57)	1.4 (0.45)	0.788
Neutral	1.3 (0.42)	1.2 (0.37)	0.900

The correlations showed significant reliabilities for most conditions in all tasks

All correlations significant at *p* < 0.05 except *

## Discussion

The previous research has shown a direct link between attentional abilities and performance on biological motion tasks (e.g., Thornton, Rensink & Shiffrar, 2002; Cavanagh, Labianca & Thornton, 2001; Chandrasekaran et al., 2010), both of which have been shown to change with age. Only in younger adults, however, has this relationship been studied. By combining the stimuli and procedure of both Cavanagh et al. (2001) and Chandrasekaran et al. (2010), the present study investigated whether age-related decline in biological motion perception is mediated by impaired attentional abilities.

### Ageing and biological motion perception

As a measure of biological motion performance, participants performed a facing direction task. As anticipated, reaction time results revealed older adults to be significantly slower than younger adults, which is in accordance with earlier studies of biological motion perception and ageing (e.g., Norman et al., 2004; Pilz et al., 2010). Contrary to our predictions, however, accuracy did not differ between age groups. This is surprising given the previous research that found older adults to be impaired in the detection and discrimination of point-light animations (Norman, et al., 2004; Billino, Bremmer & Gergenfutner, 2008; Pilz, et al., 2010; Insch et al., 2012; Spencer et al., 2016; Agnew et al., 2016). However, this finding may simply reflect the heterogeneous nature of the older adult population. In fact, our results revealed large individual differences, especially within our older group. Moreover, younger participants’ performance was overall poorer than expected when comparing it to the previous studies using similar paradigms. For example, using the same task, Pilz et al. (2010) found large age differences at 200 ms, as well a high-accuracy performance in their younger group (> 75%). Our study was relatively long, which might have led to younger adults being less motivated.

To investigate biological motion perception in relation with attentional demands, we used the same target detection task as Cavanagh et al. (2001). Participants had to detect the presence of a target point-light walker facing opposite to a varied number of distractor walkers. Overall, as set size increased, participants were less accurate and slower to respond, especially in the target-absent trials, which is in accordance with the findings of Cavanagh et al. Both age groups displayed considerable search slopes on the task; thus, our results add to the growing body of evidence suggesting that selective attention and biological motion perception share some common cognitive resources (Cavanagh et al., 2001; Thornton, Rensink & Shiffrar, 2002; Battelli, Cavanagh & Thornton, 2003; Pavlova, Birbaumer & Sokolov, 2006; Safford et al., 2010; Chandrasekaran et al., 2010). Interestingly, younger adults in this study were more accurate than those tested by Cavanagh et al. However, this accuracy advantage could be related to a speed–accuracy trade-off given that younger participants in this study were overall also slower to respond. As anticipated, age differences were found in both accuracy and reaction times. Older adults were slower and overall less accurate than younger adults, which confirms the previous research on age-related changes in biological motion perception (Norman et al., 2004; Billino et al., 2008; Pilz et al., 2010; Insch et al., 2012; Spencer et al., 2016; Agnew et al., 2016). Despite such age effects, however, there was no age-related difference in search slopes. These results indicate that the search strategies younger and older adults employed were very similar, and thus, search efficiency was comparable across age groups, regardless of response speed.

### Ageing and attentional abilities

To assess younger and older adults’ ability to selectively attend, we used both a conjunctive visual search and Stroop task (Stroop, 1935). During the conjunctive search task, participants had to indicate the presence of a target within different set sizes. In this task, the target and distracters share similarities in more than one visual property (e.g., colour and orientation). With an increase in the number of distracters, target detection becomes increasingly slowed. In addition, search times increase for target-absent trials. This was confirmed by our results, as both age groups were slower at responding to the target absent than the target-present trials, and reaction times increased with increasing set size. As anticipated, this effect was exaggerated in the older group, especially in the largest set size (e.g., Maylor & Lavie, 1998; Watson, Maylor & Manson, 2002; McCarley, Mounts & Kramer, 2004; Hommel, Li & Li, 2004). Similar to the biological motion target detection task, older adults were as efficient, albeit slower, as younger adults at searching for their targets. This is surprising and contradicts the previous studies that found older adults to exhibit steeper search slopes than younger adults (e.g., Humphrey & Kramer, 1997; McCarley, Mounts & Kramer, 2004; Hommel, Li & Li, 2004). For instance, Williams, Zacks, and Henderson (2009) tracked younger and older adults’ eye movements, whilst they performed a conjunctive search task using real-world objects. They reported that whilst both age groups displayed similar search patterns, older adults differed in the sequence in which objects were searched. Thus, it could be that our older group used a particular search sequence, which helped to compensate for their overall slowed responses.

Performance on the Stroop task (1935) was as predicted. All participants exhibited Stroop interference effects as they were slower and less accurate at responding to the incongruent colour words, compared to the congruent colour words. Performance accuracy did not differ between age groups. However, this was to be expected due to the simple nature of the task. In terms of reaction times, in comparison with younger adults, older adults were significantly slower and displayed larger interference effects, which is consistent with the past literature (e.g., Harpur, Scialfa, & Thomas, 1995; Folk & Lincourt, 1996; Humphrey & Kramer, 1997; West & Alain, 2000; Davidson, Zacks & Williams, 2003). These findings, together with the conjunctive search results, clearly demonstrate that older participants were impaired in their ability to selectively attend.

A target discrimination task was employed to measure participants’ spatial attention. Here, participants were asked to discriminate target letters (T or L), in which they had either previously been cued (valid), incorrectly cued (invalid), or not cued at all (neutral) to their location. Commonly, participants are slower and less accurate at responding in the invalid than in the valid and neutral trials (Posner, 1980), which was confirmed by our results. As expected, this effect was more pronounced in the older group, which aligns with the previous studies (Madden, 1992; Madden, Connolly & Pierce, 1994; Faust & Balota, 1997). In fact, older adults were slower to respond than younger adults across all cue conditions.

An advantage in accuracy for older adults on both the conjunctive search and spatial cueing task was unexpected. Overall, older adults were better than younger adults at identifying and discriminating the targets on both tasks (present trials only for the conjunctive search). This advantage for older adults contradicts the previous studies, which either found no accuracy differences between age groups (Greenwood, Parasuraman, Haxby, 1993; Foster, Behrmann, Stuss, 1995; Scialfa & Joffe, 1997; Tricks & Enns, 1998; Greenwood & Parasuraman, 2004; Gottlob, 2006), or found younger adults to be more accurate on both spatial cueing and conjunctive search tasks (Folk & Hoyer, 1992; Lincourt, Folk & Hoyer, 1997; McCalley, Bouwhuis & Juola, 1995; Humphrey & Kramer, 1997). A speed–accuracy trade-off might explain the advantage for older adults as they took longer to respond than younger adults. Another explanation might be motivational factors. Ageing is typically associated with declining cognitive abilities, and it has been suggested that such negative association motivates older adults under certain conditions. For example, Ennis, Hess, and Smith (2013) found older adults to be highly motivated and more effortful than younger adults on a range of cognitive tasks, and suggested that this was due to older adults wanting to perform as well as younger adults.

### The relationship between age, biological motion processing, and attentional abilities

To assess the relationship between attentional abilities, biological motion perception, and age, we computed correlations on both accuracy and reaction time data. Surprisingly, we found no significant correlations between reaction time, or accuracy performance across all the biological motion and attention tasks for either age group. Our results that age, biological motion processing and attentional abilities are not related, is surprising given the correlational findings of Chandrasekaran et al. (2010). In their study, performance on the biological motion task inversely correlated with the amount of interference younger participants exhibited on the Stroop task. There were subtle differences in how Chandrasekaran et al. and the current study recorded responses in the Stroop task (1935). Whilst Chandrasekaran et al. manually recorded their participants’ answers, we recorded participants’ responses via key presses. Nevertheless, large Stroop interference effects were found in both studies. Therefore, it is unlikely that these response differences were driving the non-correlational findings in our study.

Another difference between the present study and Chandrasekaran et al. (2010) is that their biological motion action discrimination task required participants to discriminate the orientation of a wide range of upright and inverted point-light actions. Contrastingly, our biological motion task required only the discrimination of the facing direction of upright tennis players. It has been suggested that the discrimination of inverted actions requires global integration across time, whereas the discrimination of upright actions can be solved solely using local cues (Thornton & Vuong, 2004; Shi, Weng, He & Jiang, 2010; Thompson & Parasuraman, 2012). Furthermore, with regard to the target detection task, studies have shown that the discrimination of direction of walkers is possible from local cues (Troje & Westhoff, 2006; Spencer et al., 2016). Therefore, it is possible that the nature of our biological motion tasks might not have engaged in active attentional processes that were necessary to do the orientation discrimination task in Chandrasekaran et al. Thus, the differences in tasks and stimuli between studies might provide an explanation for our lack of significant correlational findings between the Stroop and the biological motion discrimination task.

Interestingly, the previous studies on younger and older adults have shown that visual perceptual tasks are not necessarily related; therefore, our lack of significant correlations may not be as surprising. For example, Shaqiri, Clarke, Kunchulia, Herzig, Pilz & Herzog (2015) compared younger (108) and older (131) adults’ performance on 14 different perceptual tasks (e.g., motion perception, orientation sensitivity, and biological motion perception) and four cognitive tasks (e.g., MoCA and digit span). Importantly, they did not find many relevant significant correlations between the different measures. Similarly, Agnew et al. (2016) compared younger and older adults’ performance on a biological motion-matching task with performance on the Navon task (Navon, 1977), and found no relationship between the measures. Furthermore, non-significant correlations between our different attention tasks, in line with the previous studies that found different measures of global/local processing, were not related. For instance, Chamberlain, Van der Hallen, Huygelier, Van de Cruys and Wagemans (2017) tested over 250 younger participants on three measures that are often taken as measures for local and global processing biases: coherent motion processing, the embedded figures test, and the Navon task. They did not find significant correlations in samples of above 250 participants. In addition, Dale and Arnell (2013) compared younger participants’ performance on three distinct global/local measures (Navon letters, hierarchical shapes, spatial frequency faces), and also found no relationship between the three measures.

Our results are in line with research on younger adults that has found no correlations between tasks that are commonly thought to be related. Cappe, Clarke, Mohr, and Herzog (2014), for example, were the first to illustrate the absence of a common factor underlying visual perceptual tasks. They tested students on a battery of visual paradigms (e.g., visual acuity, Gabor detection, and vernier discrimination), and found few significant correlations between the different visual measures. Furthermore, Grzeczkowski, Clarke, Francis, Mast, and Herzog (2017) found no relationship between the perception of different visual illusions (e.g., Ebbinghaus and Ponzo), which suggests that there is no common factor for the perceptibility for visual illusions. Our findings, therefore, add to this growing body of evidence, suggesting that individual performance across tasks cannot be pinned down to one single factor. Certainly, our age groups demonstrated large variability within their performance across all tasks. However, whereas our participants may have performed well in one biological motion tasks, it did not relate to their performance in the other biological motion task. Moreover, we showed that age-related changes in biological motion perception are unrelated to changes in attentional performance.

### Conclusion

Taken together, the results of the present study are the first to illustrate that age-related changes in biological motion perception are not mediated by impaired attentional abilities. Older adults were significantly slower, and generally worse at discriminating point-light targets, compared to younger adults. Also found, were age-related deficits in visual attention, hallmarked by increasingly slowed responses in older adults, across all attention tasks. However, these age differences were found not to be related. In addition, our results add to the growing body of evidence suggesting that selective attention and biological motion perception share some common cognitive resources. Decreased biological motion processing can affect many aspects of older adults’ daily lives; importantly, however, our results suggest that attention plays a limited role in this decrement.
